# TELS: Evolution patterns of research keywords from the evidence of PNAS Social Sciences topics

**DOI:** 10.3389/fdata.2022.1045513

**Published:** 2022-11-10

**Authors:** Bing Liu, Mengfan Shi, Yi Kuang, Xin Jiang

**Affiliations:** ^1^LMIB & School of Mathematical Science, Beihang University, Beijing, China; ^2^Zhengzhou Aerotropolis Institute of Artificial Intelligence, Zhengzhou, China; ^3^LMIB & NLSDE & Institute of Artificial Intelligence, Beihang University, Beijing, China; ^4^Zhongguancun Laboratory, Beijing, China

**Keywords:** keyword analysis, keywords evolution pattern, log-normal distribution, keywords novelty, elite keywords

## Abstract

By reviewing scientific literature, researchers may obtain a comprehensive understanding of field developments, keeping abreast of the current research status and hotspot shifts. The evolution pattern of keywords is supposed to be an efficient indicator in revealing the shifting and sustainability configuration of scientific concepts, ideas, and research hotspots. Here we take an extensive investigation of the evolution of keywords among all publications in PNAS Social Sciences from 1990 to 2021. Statistical tests show the keyword mention time series always accompanied by the emergence of a log-normal distribution. Additionally, we introduce a novel schema of four patterns (TELS), which are Transient impact type, Explosive impact type, Large impact type, and Small impact type, respectively, to illustrate the evolution of keywords. The TELS schema can be used to capture the whole life circle feature of any proposed keyword, from a pool of candidates. By dividing the entire time into four periods, we also introduce the concept of elite keywords to reveal the temporal feature of social sciences focus. An explicit transition from anthropology research to neuroscience and social problems research can be observed from the evolution diagram. We argue that the proposed method is of general sense and might be applicable to other fields of science.

## 1. Introduction

Reviewing sufficient scientific literature can help researchers obtain a comprehensive understanding of field developments and hotspot shifts, and may accelerate the corresponding cross-field research. Generally, the keywords of a scientific literature are closely related to the main content of the corresponding research (Zhang et al., 2018), thus well reflect the research topics. The life cycle of a keyword is associated with the significance of the keyword itself and its impact on the field. Discovering the evolutionary mode of keywords may help researchers to find out the research development and the frontiers of the field. In this sence, author-selected keywords are considered a significant channel of scientific concepts, ideas, and knowledge (Cobo et al., 2011).

At present, the frontier research and disciplinary development exploration of scientific research based on academic literature data has become quite an active field (Chen et al., 2016). Numerous studies have started from the experience of domain experts and use a priori knowledge as a guide to conduct research and discoveries in the scientific literature (You et al., 2021). We also note that several fascinating studies use various forms of keyword analysis such as co-occurrence network to mine the domain knowledge based on bibliometrics (Su and Lee, 2010; Dehdarirad et al., 2014; Radhakrishnan et al., 2017). These domain knowledge discovering methods basically belong to quantitative analysis and are usually supported by exact mathematical models or theories.

Recently, a great number of scholars have focused on word frequency analysis, co-word analysis, and co-occurrence networks combined with scientific knowledge graph technology to analyze research hotspots and future research trends in different fields. For instance, Ohniwa et al. (2010) apply co-word analysis combined with a knowledge map to reveal the evolution trend of life sciences in the past 30 years and discover the emergence and development of emerging disciplines. Duvvuru et al. (2013) propose a keyword network model in academic publications which can be used to discover academic research trends. Besides, the organization and evolution patterns of keywords can be also revealed through statistical analysis, visualization of network structure, and temporal characteristics. Madani and Weber (2016) take bibliometric analysis and keyword-based network analysis to identify the top researchers in the field of patent mining, and the clustering in the keyword network shows three main stages of patent mining evolution. González et al. (2018) perform keyword statistical analysis and network analysis to discover the development of the field of sports sciences category included in the Web of Science database (WoS database), they analyze the survival time of new words that have appeared since 2001 and built a co-occurrence network between words to find 6 large topic clusters. Some other studies discuss keywords from a visualization perspective. Ho et al. (2021) performed bibliometric analysis and identified key national contributors and international coalitions and shifts in field hotspot research over the past 25 years, by applying visualization software VOS and bibliometrix software package based on WoS database.

In this study, we consider the keywords as a nice indicator to understand the evolution of research interests. In fact, keywords in different domains may perform various time evolution patterns. However, few works have been done to study and demonstrate the form of these time-evolving patterns. Moreover, most keyword analysis research focuses on articles indexed on WoS in certain fields, so the data obtained for articles are often incomplete. In this paper, we collect keywords information from all the publications in the database of Proceedings of the National Academy of Sciences (PNAS) Social Sciences. We propose a schema TELS containing four patterns to describe the evolution pattern of keywords. We also analyzed the distribution of mention times and demonstrate that most of them can be characterized by a log-normal distribution. We point out that the novelty of keywords has a universal decay characteristic. Finally, by defining elite keywords based on the Term Frequency-Inverse Document Frequency (TF-IDF) value, we reveal the changes in research hotspots and directions of social sciences in each era from 1990 to 2021.

## 2. Data and methods

In this section, we mainly introduce the data set and the analysis methods used in this research. Based on the collected data set, we further investigate the keywords evolution pattern of publications in PNAS Social Sciences topic between 1990 and 2021.

### 2.1. Publications data

By designing a python crawler program, we get data from the social sciences section of the PNAS's official website: https://www.pnas.org/. The data set we collected contains relevant information of all publications under social sciences category from 1990 to 2021, including the article title, keywords, publication date, article type, and the subsegment field of the article. After some preprocessing operations, such as removing reduplication or supporting publications, and publications which have no keywords, a data set containing of 2, 935 publications is obtained.

### 2.2. Keyword data overview

Basically, keywords are important carriers of scientific concepts, ideas, and knowledge (Tijssen and Van Raan, 1994). We extracted 7, 246 unique keywords from the above mentioned 2, 935 publications with 12, 737 frequencies. The average number of keywords per article is found to be 4.3. About 58% articles have five keywords, 21% articles have four keywords, 16% articles have three keywords, < 5% of articles have keywords less than two or more than seven.

### 2.3. Mention time series

**Figure 1:**
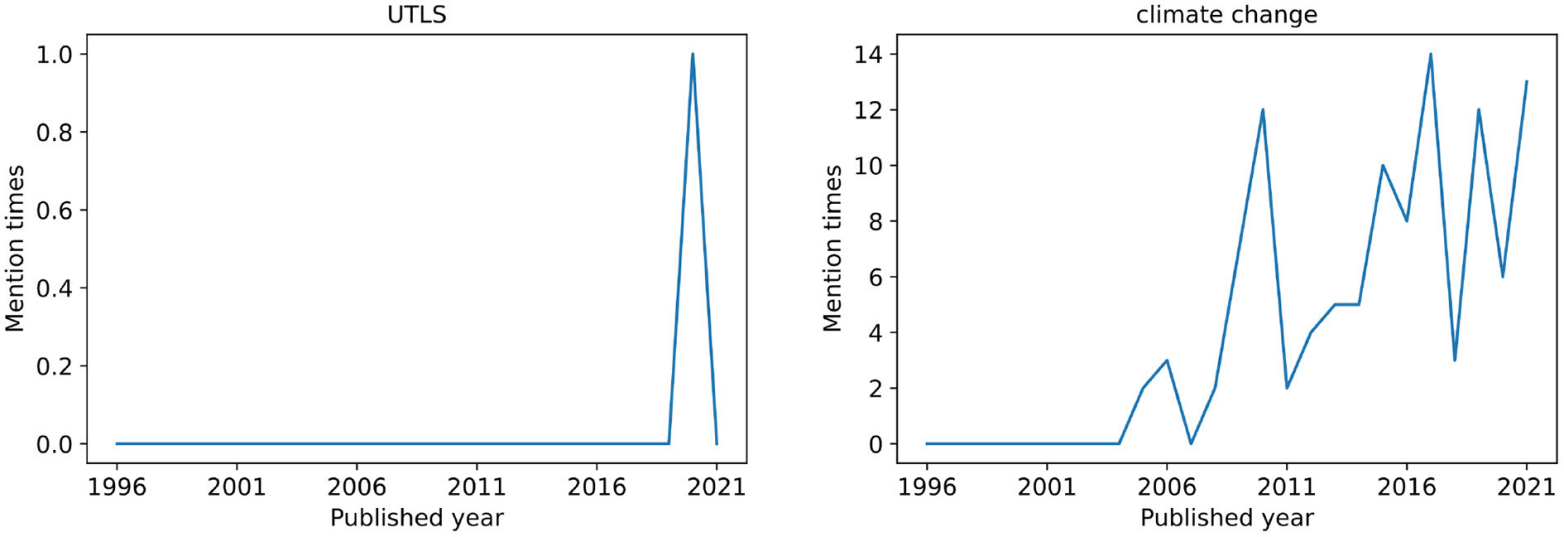
Examples of keyword mention time series. Among all articles of PNAS social sciences from 1990 to 2021, only one article used the word UTLS as a keyword. The word climate change has been designated as a keyword by articles for many times since 2005, and it has been maintained in a relatively popular state.

In order to study the life law of the keywords changing with time, here we introduce the concept of mention time series of keywords, which is the number of mentions for each keyword during the considered time period. For each keyword, we try to find different patterns in the temporal variation of the keyword mention time series. Concretely, the mentioned times of each keyword in each year is recorded. To illustrate, we show the mention times of keyword UTLS and climate change from 1990 to 2021 in Figure 1.

### 2.4. Time period of data

We divided the entire period into four parts, 1990–1999 classified as 1990s, 2000–2009 as 2000s, 2010–2019 as 2010s, and 2020–2021 as 2020s. The total number of keywords appearing in each era and the total number of articles in the entire corpus are shown in the Table 1. In the 1990s, the dataset contained 20 articles and 68 different keywords. In the 2000s, it contained 440 articles and 1, 369 different keywords. It can be clearly seen that after entering the 2000s, the number of keywords has risen sharply, and the collection of articles has also expanded significantly. By the 2010s, it contained 1, 817 articles and 5, 028 different keywords. The 2020s part contains year 2020 and 2021, with 658 articles and 2, 080 unique keywords appearing in this period.

**Table 1:** The total number of articles in each era and the number of keywords included.

	**1990s**	**2000s**	**2010s**	**2020s**
The range of time	1990–1999	2000–2009	2010–2019	2020–2021
Unique keywords	68	1,369	5,028	2,080
All keywords	75	1,752	8,002	2,908
Articles in the corpus	20	440	1,817	658

### 2.5. K-means clustering algorithm

The k-means clustering algorithm is an unsupervised machine learning technique used to identify clusters of data sets. This algorithm was independently proposed by Lloyd (1957), Ball and Hall (1965), MacQueen (1967), and Hartigan and Wong (1979) in their respective different scientific research fields. The most common k-means method is the Lloyd algorithm (Lloyd, 1982), which works well when the clusters are dense and the differences between the clusters are obvious.

The Lloyd algorithm for k-means clustering is an iterative process. The purpose of iteration is to minimize the sum of squares (SSE) of the distance from all samples in the cluster to the cluster center. Generally, this clustering algorithm contains the following four steps. First, choose *k* initial cluster centers and randomly partition the data into *k* sets. In practice, here we use the k-means++ method (Vassilvitskii and Arthur, 2006) in the python library to select the initial cluster center. This method proposes a practical randomized initialization of the centers. Second, calculate the center of each set. Each center is updated to minimize its average distances to the points assigned to it. The optimal center is believed to be the mean or median of the points in each category. Third, each point is assigned to the center that is closer to it. Last, repeat the last two steps until no point can be moved, or the maximum iteration number is reached.

In the first step of the Lloyd algorithm, the value of the cluster *k* needs to be specified in advance. Here, we utilize the elbow method (Thorndike, 1953) to get the optimal *k* value. An important indicator of the elbow method is the sum of the squared errors (SSE),


(1) $$\begin{array}{l} \text{SSE}=\overset{k}{\underset{i=1}{\sum }}\underset{p\in C_{i}}{\sum }|p-m_{i}{|}^{2}, \end{array}$$

where *C*_*i*_ is *i*-th cluster, *p* is the point in *C*_*i*_, *m*_*i*_ is the centroid of *C*_*i*_. SSE is the clustering error of all samples, which represents the quality of the clustering effect.

The core idea of the elbow method is that the decreasing of SSE will encounter a point of inflection as *k* increases, as shown in the upper panel of **Figure 3A**. As *k* is increasing to the real number of clusters, the decline of SSE is supposed to be rapid. However, after *k* reaches the true number of clusters, the decline of SSE will become flat as *k* continues to increase. In this sense, the relationship between SSE and *k* looks like a shape of an elbow, and the inflection point value corresponding to the so-called elbow point is considered to be the true number of clusters of the data.

After obtaining the value of cluster *k*, for a given dataset *X* = *x*_1_, *x*_2_, ...., *x*_*j*_, ...., *x*_*n*_ containing *n* *d*-dimensional data points, where *x*_*j*_ belongs to *S*_*i*_(*i* = 1, 2, ..., *k*). The Lloyd algorithm takes a set of data and clusters them into *k* partitions. Each partition represents a class *S*_*i*_, and each class *S*_*i*_ has a class center μ_*i*_. Select Euclidean distance as similarity and distance judgment criteria, the within-cluster sum of squares *J* is minimized iteratively:


(2) $$\begin{array}{l} J=\overset{k}{\underset{i=1}{\sum }}\underset{x_{j}\in S_{i}}{\sum }|x_{j}-\mu_{i}{|}^{2}, \end{array}$$

where μ_*i*_ is the mean of all the points in cluster *S*_*i*_.

### 2.6. Kolmogorov-Smirnov (KS) test

The KS test is a nonparametric hypothesis test in statistics used to test whether a single sample obeys a certain distribution, or whether two samples obey the same distribution.

In the case of a single sample, we want to test whether the sample obeys a certain distribution function *F*_0_(*x*), and specify *F*_1_(*x*) as the empirical distribution function of the sample. We have assumptions: *H*_0_:*F*_0_(*x*) = *F*_1_(*x*), *H*_1_:*F*_0_(*x*)≠*F*_1_(*x*). The KS statistic is constructed as : *D*_*n*_ = *max*_*x*_|*F*_1_(*x*) − *F*_0_(*x*)|. In fact, the KS statistic describes the distance between the empirical distribution function of a sample distribution and the cumulative distribution function of a reference distribution, or the distance between the empirical distribution functions of two sample distributions. The KS statistic takes value between 0 and 1.

The critical value of the KS statistic with 99% confidence $\bar{D_{n}}$ is shown as Table 2, *n* is the number of samples. If the value of the KS statistic we get from the sample is less than $\bar{D_{n}}$, we accept the null hypothesis, otherwise, we reject the null hypothesis.

**Table 2:** At the significance level of 0.20, 0.15, 0.10, 0.05, 0.01, the critical value of the KS test *D* statistic, *n* is the sample size (Massey, 1951).

***n***	$\bar{D_{n}}$					***n***	$\bar{D_{n}}$				
	**0.20**	**0.15**	**0.10**	**0.05**	**0.01**		**0.20**	**0.15**	**0.10**	**0.05**	**0.01**
1	0.900	0.925	0.950	0.975	0.995	13	0.284	0.302	0.325	0.361	0.433
2	0.684	0.726	0.776	0.842	0.929	14	0.274	0.292	0.314	0.349	0.418
3	0.565	0.597	0.642	0.708	0.828	15	0.266	0.283	0.304	0.338	0.404
4	0.494	0.525	0.564	0.624	0.733	16	0.258	0.274	0.295	0.328	0.392
5	0.446	0.474	0.510	0.565	0.669	17	0.250	0.266	0.286	0.318	0.381
6	0.410	0.436	0.470	0.521	0.618	18	0.244	0.259	0.278	0.309	0.371
7	0.381	0.405	0.438	0.486	0.577	19	0.237	0.252	0.272	0.301	0.363
8	0.358	0.381	0.411	0.457	0.543	20	0.231	0.246	0.264	0.294	0.356
9	0.339	0.360	0.388	0.432	0.514	25	0.21	0.22	0.24	0.27	0.32
10	0.322	0.342	0.368	0.410	0.490	30	0.19	0.20	0.22	0.24	0.29
11	0.307	0.326	0.352	0.391	0.468	35	0.18	0.19	0.21	0.23	0.27
12	0.295	0.313	0.338	0.375	0.450	Over 35	$\frac{1.07}{\sqrt{n}}$	$\frac{1.14}{\sqrt{n}}$	$\frac{1.22}{\sqrt{n}}$	$\frac{1.36}{\sqrt{n}}$	$\frac{1.63}{\sqrt{n}}$

### 2.7. Elite keyword definition

We consider a word to be an period elite keyword when its mention is dominant in a certain period and relatively rare throughout the period. An intuitive idea is to find the keywords with the most occurrences, which means if a keyword is essential, it should appear in a majority of articles in the period.

Generally, Term Frequency (TF) is used to measure that how many times a term is present in a document. It is necessary to penalize words that appear multiple times in each era. Thus, Inverse Document Frequency (IDF) is introduced to assign lower weight to frequent words and assign greater weight for the words that are infrequent. The multiplication of TF and IDF forms TF-IDF. Actually, TF-IDF is often used as a numerical statistic indicator that shows the relevance of keywords to some specific corpus (Qaiser and Ali, 2018).

To proceed this study, we use TF-IDF to identify whether a keyword is an elite one or not. TF-IDF is proportional to the mention times of a keyword in a certain era and inversely proportional to the number of articles containing that keyword in all periods.


(3) $$\begin{array}{l} \text{TF-IDF}=\text{TF}*\text{IDF}. \end{array}$$

Here TF is obtained by calculating the frequency of each keyword appearing in each era. Considering the divergence of the total number of keywords, we normalized the word frequency TF as


(4) $$\begin{array}{l} \text{TF}=\frac{TF_{k}}{TF_{total}}, \end{array}$$

where *TF*_*k*_ is the number of mention times of a keyword *k* in a certain era, and *TF*_*total*_ is the total mention times of all the keywords that appear in a certain era.

Second, we calculate the IDF of a keyword. In this situation, we consider the keywords of all publications as the corpus, which is used to represent the use environment of the language. If a keyword is more common, the IDF should be much smaller and closer to 0.


(5) $$\begin{array}{l} \text{IDF}=log\left(\frac{NP}{NP_{k}+1}\right), \end{array}$$

where the *NP* is the number of publications in the corpus, the *NP*_*k*_ is the number of publications containing the keyword *k*. The purpose of adding 1 to the number of articles containing the keyword *k* is to prevent the denominator becoming 0.

## 3. Results

In this section, we study domain keywords from three perspectives. First, we propose a novel schema of four patterns, which are Transient impact type, Explosive impact type, Large impact type, and Small impact type respectively, to illustrate the evolution of keywords. Second, the statistical tests for the most keywords show the occurrence of a log-normal distribution. Finally, by dividing the entire time into four periods, we introduce the concept of elite keywords to reveal the temporal feature of social sciences focus.

### 3.1. Cluster analysis of mention time series

We carefully observe the mention time series of keyword and find that various patterns emerges during the evolution. To proceed, we take some cluster analysis of these time series. Four typical patterns are demonstrated in the clustering results.

#### 3.1.1. Dynamic time series data clustering

Time series clustering has been extensively investigated. The clustering features are believed to be significant in mining underlying patterns of massive fragments of time series. One renowned heuristic method for crisp partitions of time series is the k-means clustering algorithm (MacQueen, 1967). Here we execute k-means clustering on the low-dimensional feature embedded vectors of the original time series. These low-dimensional features are discussed in details in the following part.

#### 3.1.2. Feature extraction

**Figure 2:**
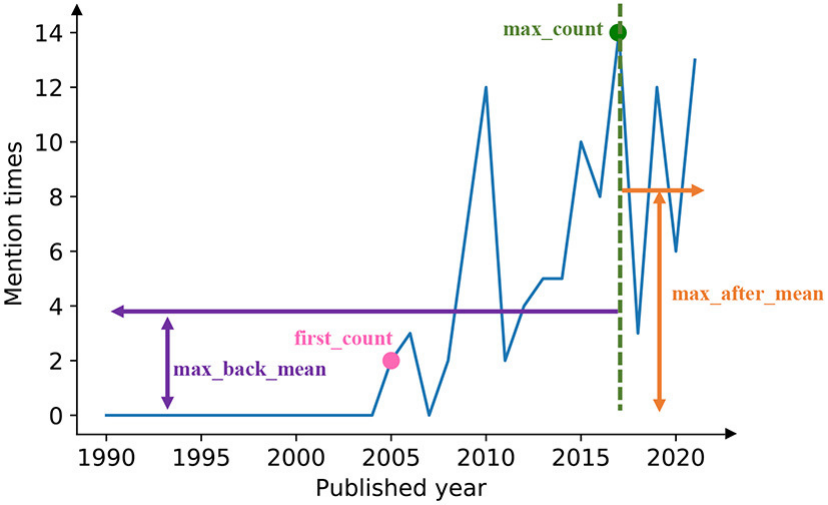
Four characteristic numbers defined for time series. For each mention time series, we extracted four characteristics to represent the time series, as shown in the figure: max_back_mean, first_count, max_count, max_after_mean.

To get some static and low-dimensional features of the mention time series, we introduce four features to embed each time series into a four-dimensional vector (Figure 2). The four components of the feature vector are named as first_count, max_count, max_back_mean, and max_after_mean, respectively.

- First_count: the mention times of the keyword in the year when the word first appears.
- Max_count: the maximum mention times for a keyword in 1 year during the whole period.
- Max_back_mean: the average mention times of the keyword over the years before reaching the max_count.
- Max_after_mean: the average mention times of the keyword over the years after reaching the max_count.

#### 3.1.3. Clustering results

In this form, for each keyword, the corresponding mention time series represented by four characteristics. To generize, we take the typical z-score normalization form of the vector, which is,


(6) $$\begin{array}{l} x^{*}=\frac{x-\mu }{\delta }, \end{array}$$

where μ represents the mean value of each characteristic, δ is the standard deviation.

**Figure 3:**
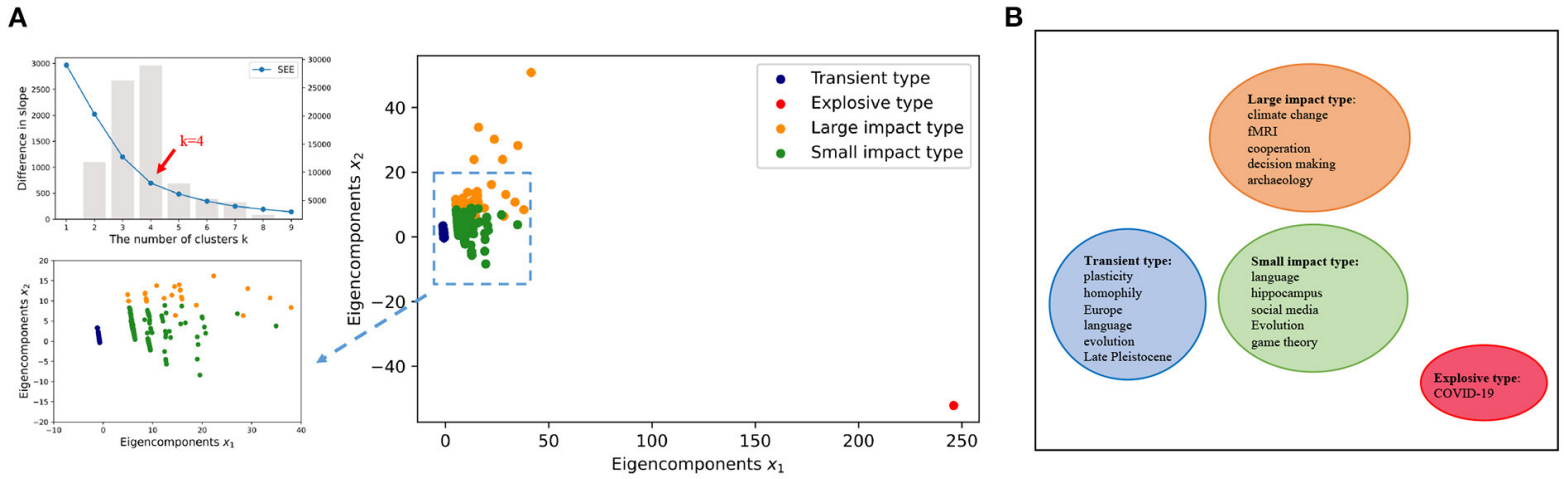
Data mapping. **(A)** Minimize the SSE and use the elbow method to select the optimal number of clusters *k* = 4. We use k-means clustering algorithm to divide 7, 246 keywords into four clusters, and the data is projected into a low-dimensional space using LDA. The LDA algorithm is a dimensionality reduction technique for supervised learning, which is based on the smallest intra-class variance and the largest inter-class variance after projection. **(B)** Subsequently, keywords with high frequencies in each cluster are used to represent the cluster.

During the clustering, the optimal number of clusters *k* = 4 is obtained by minimizing the SSE (Figure 3A). Seven thousand two hundred and forty-eight unique keywords are clustered into four groups using the k-means clustering algorithm (Lloyd, 1982). To illustrate, the four clusters are also projected into a two-dimensional space by the Linear Discriminant Analysis (LDA) (Balakrishnama and Ganapathiraju, 1998). To make a first glance of the clusters, we also select five keywords with frequently mention times in each group to represent the mainsteam topic of the cluster (Figure 3B).

**Figure 4:**
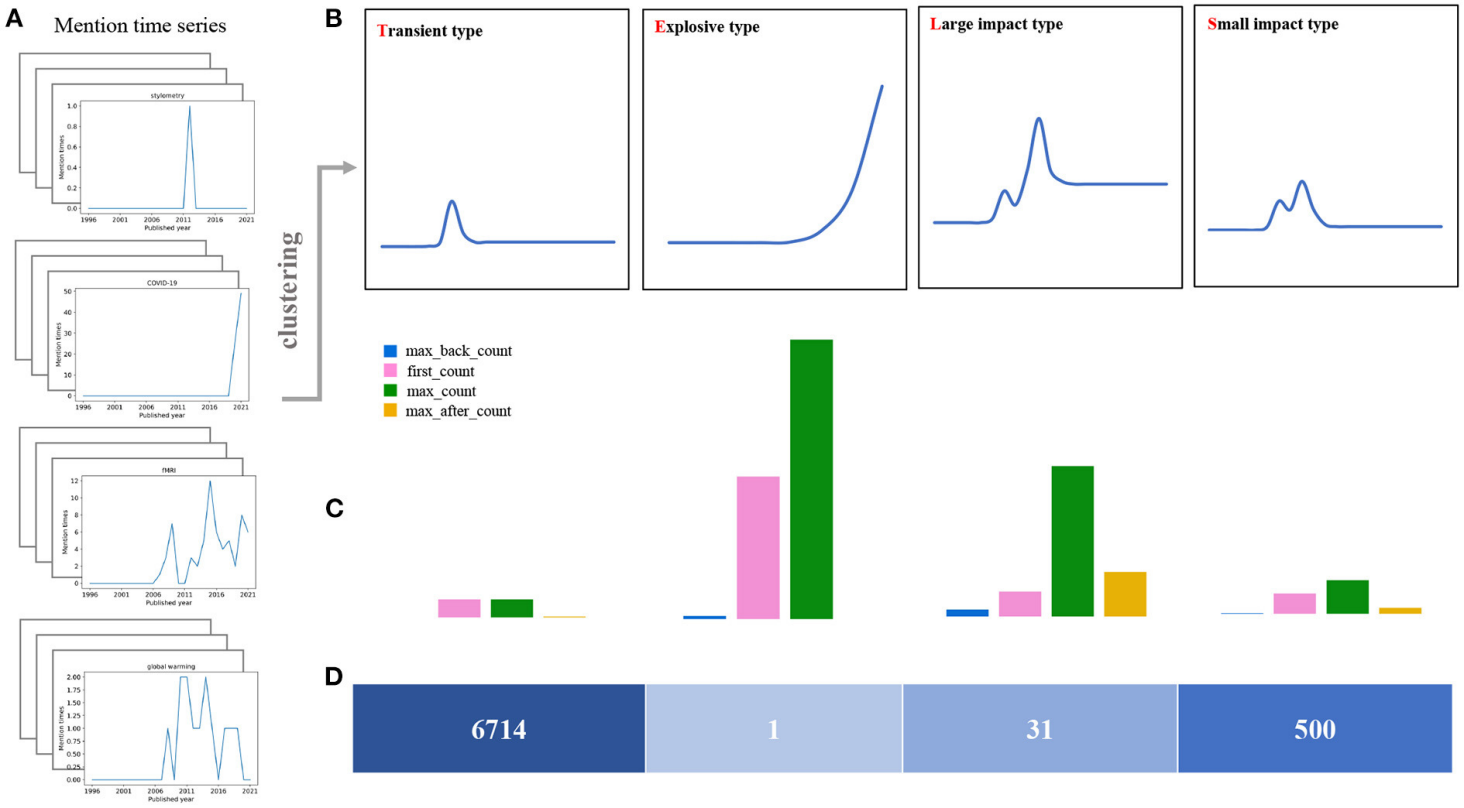
Cluster analysis of mention time series. **(A)** Four keyword mention time series examples in the data set. **(B)** The average mention time series for each cluster, which corresponds to the four keyword evolution patterns present in the dataset. **(C)** Cluster centroids are plotted as bars, and the height of the vertical axis corresponds to the value of each feature. **(D)** The number of keywords in each cluster.

Based on the clustering results as shown in Figure 3, we argue there exist four typical patterns in the keyword mention time series. These patterns indicate four evolution configurations of the keyword's impact on the subject. In Figure 4, we demonstrate these four patterns as the TELS pattern. To make a direct impression, we display four typical keywords examples in Figure 4A, the average mention time series per cluster in Figure 4B, the cluster centroids represented as a bar diagram in Figure 4C, and the number of keywords within each cluster in Figure 4D.

**Figure 5:**
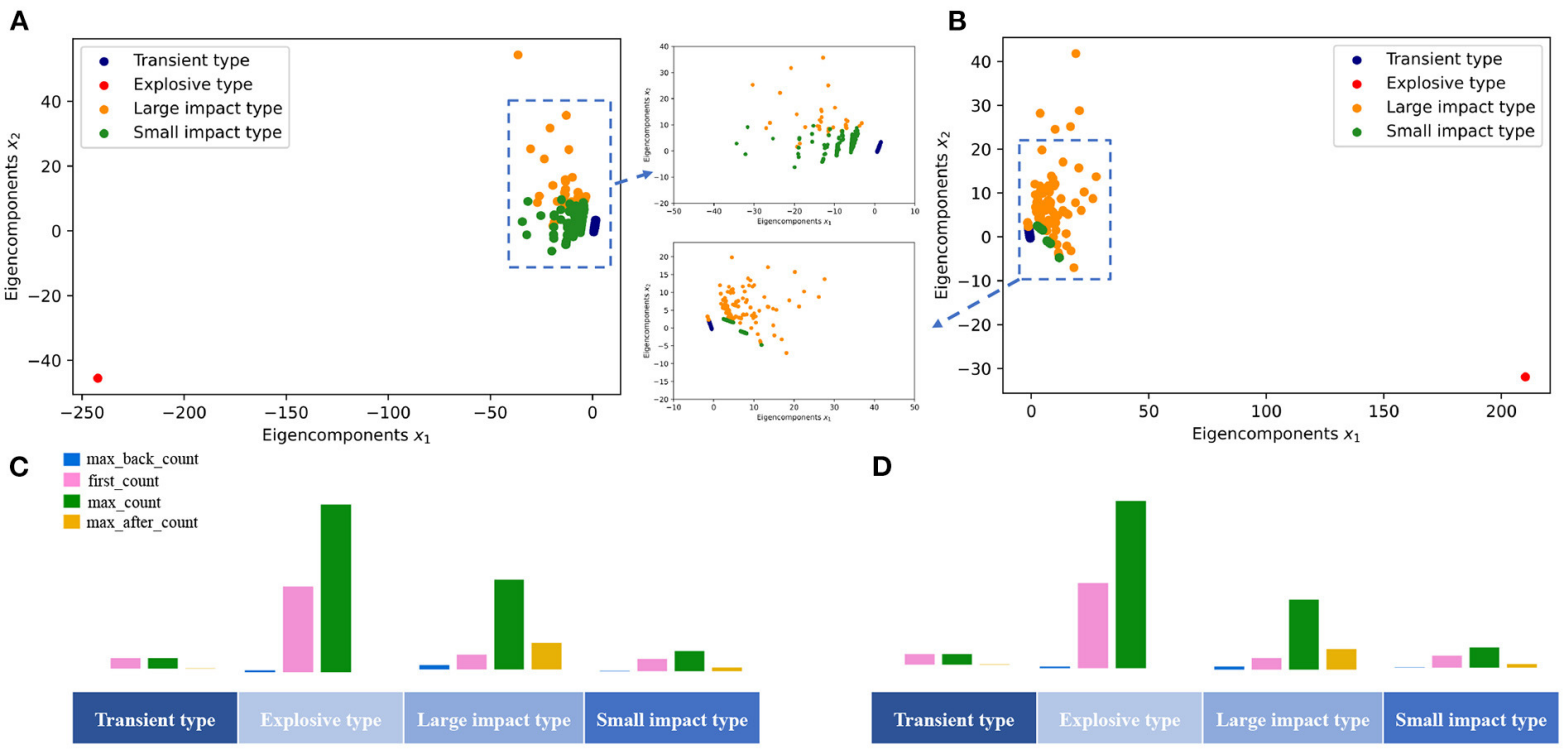
Results of clustering using two other classical clustering methods. **(A)** Agglomeration clustering results. **(B)** GMM clustering results. **(C)** Distribution of each cluster with Agglomeration clustering. **(D)** Distribution of each cluster with GMM clustering.

To verify our results, we also perform another two classic cluster analysis methods, the hierarchical Agglomeration clustering model (Murtagh and Contreras, 2012), and Gaussian Mixed Model (GMM) (Reynolds, 2009), to the data set. The corresponding results are shown in Figure 5. It is demonstrated that all these clustering methods indicate the same result that the mentioned time series can be divided into four groups.

1. **Transient type (T)**: Ninety percent of the keywords belong to this cluster or called as pattern, and the keywords within this cluster have little following and barely fluctuating mentions. From the within-class mean, the average of the highest mentions is only a bit larger than 1. The average number of mentions before reaching the maximum is nearly 0, and the average number of mentions after reaching the maximum of mentions is only 0.0357. In fact, most keywords in this category have only appeared once in the long history, or are rarely mentioned. We consider most of these keywords might have attracted relatively less interests during the development of the social sciences field. The corresponding topics have not received widespread and long-term attention, and become fleeting in the history. Generally, these keywords contain words or abbreviations related only to some certain year, or some unfashionable research directions, such as UTLS, Zika, Croatia, and Late Pleistocene.
2. **Explosive type (E)**: Keywords in such a cluster have received explosive amount of attention recently and rapidly. This cluster contains only one keyword: COVID-19. This pattern is quite different from the other three, which behaves like an exponential function. In fact, the keyword COVID-19 have attracted massive attentions and discussions in the past two years in the field of social sciences. We also notice that the number of maximal mention times of this keyword has passed 49 in 2021, and the related article number is over 84 within only 2 years, which has never happened in the past. In this sense, we argue keywords of this pattern will still rise significantly in the near future and can be regarded as a considerable hot spot and popular topic in the research field.
3. **Large impact type (L)**: Keywords in this cluster have received a long time lasting attention. For instance, this cluster contains 31 words, 26 of which are in the top 50 words with most mention time frequency. Basically, these keywords have received significant attention in the field for a long time and have a high attention intensity. The average number of mention times is 6.323, and the average number of mention times after reaching the max mentions is 2.923, which is also the highest among the four categories. We argue these keywords are mostly the core interest and hotspots of the research in the field, such as climate change, fMRI, cooperation, social networks, et al..
4. **Small impact type (S)**: Keywords in such cluster have lower volatility and relatively smaller mention times compared with the Large impact type. This cluster contains about 500 words, which also receive long-term attentions from researchers. However, they are significantly less intense than the Large impact type. This result is mainly reflected in the average of the highest number of mentions. The average of the highest number of mentions in this category is 3.148, which is much lower than 6.323. The difference is also reflected in the average number of mentions after the highest mention, which is 0.129575, considerably lower than the 2.923. The keywords in this category do not have a strong influence on the field, but the influence still maintains for a long time. It mainly includes some long-standing social phenomena or social problems, for example, language, global warming, gender differences, et al..

In total, among all the keywords, more than 90% of the keywords belong to the Transient type. This type has shown a brief peak during the long-term history, then declined to lower mention levels. A large part of the remaining keywords belongs to the Small impact type. The main difference is that, keywords of the Transient type disappear soon after their occurrence, while keywords in the Small impact type still attract some attentions in a long lasting term. The evolving pattern of Large impact type, consisting of a small subset of elite words, is some kind similar to the Small impact type, except that this type usually received extensive attention from researchers. The last type of cluster is the Explosive type. This type of word is the most concerned direction in the field in recent years, and is very popular and the mention times continues rising.

### 3.2. Distribution of keywords

In this section, statistical test results based on T, L, and S pattern data show that the keyword mention time series are following log-normal distributions. It is demonstrated that the log-normal distribution of mention times can be explained by the novelty factor model (Wu and Huberman, 2007).

#### 3.2.1. Distribution of keywords

We reveal the properties of an evolutionary process by determining the cumulative statistical distribution of the number of mentions throughout its evolution. By summing up the mention frequencies over time, we obtain the cumulative number of keywords mentions *N*_*k*_ (*t*_*i*_) of a keyword *k* for any time frame *t*_*i*_ (Asur et al., 2011),


(7) $$\begin{array}{l} N_{k}\left(t_{i}\right)=\overset{i}{\underset{\tau =1}{\sum }}n_{k}\left(t_{\tau }\right), \end{array}$$

where *n*_*k*_(*t*_τ_) is the number of mentions on keyword *k* in time interval *t*_τ_.

Then, we calculate the ratio of cumulative mentions *C*_*k*_(*t*_*i*_, *t*_*j*_) for keyword *k* for time frames *t*_*i*_ and *t*_*j*_,


(8) $$\begin{array}{l} C_{k}\left(t_{i},t_{j}\right)=\frac{N_{k}\left(t_{i}\right)}{N_{k}\left(t_{j}\right)}, \end{array}$$

where *t*_*i*_ > *t*_*j*_.

**Figure 6:**
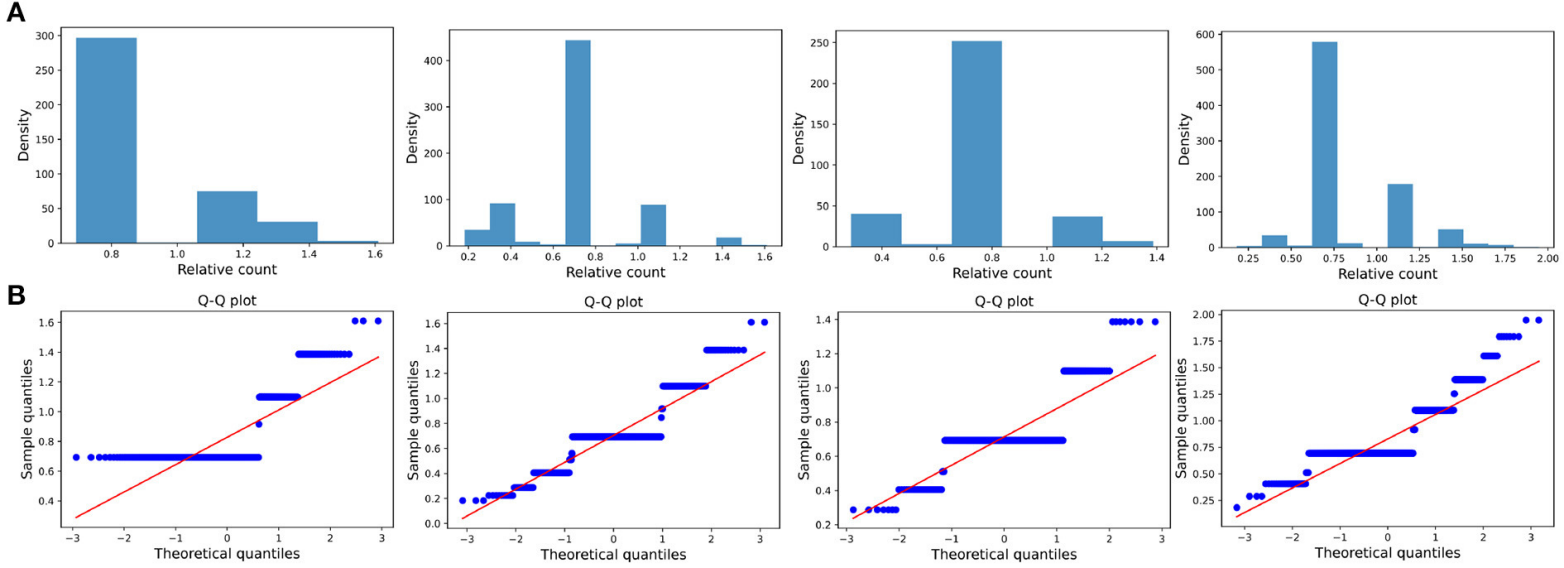
Transient type. **(A)** The ratio density of cumulative mentions for the four time intervals. From left to right in the figure, the indicators of the time intervals are: (2000, 2015), (2000, 2020), (2010, 2015), and (2010, 2020), and the x-axis is logarithmically scaled. **(B)** The Q-Q plots of the distribution of cumulative mentions relative to the normal distribution. If the random variable of the data is a linear transformation of a normal variable, the points will line up on the straight line shown in the plots.

**Figure 7:**
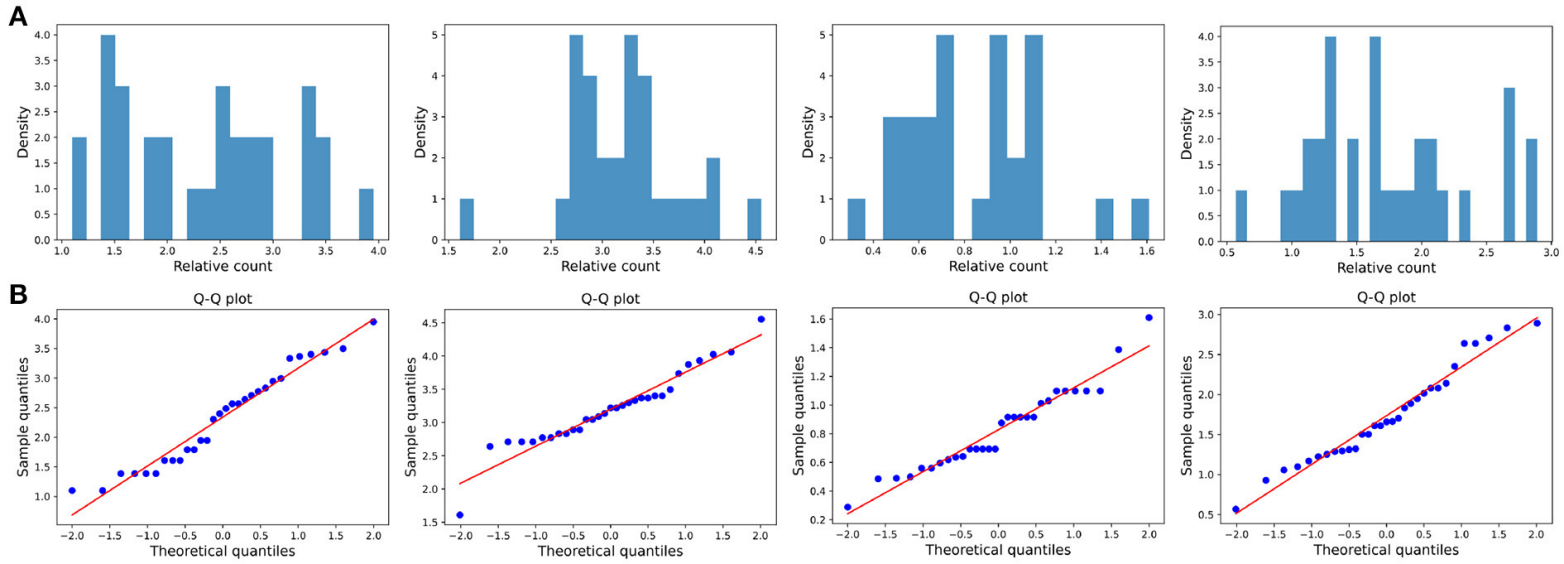
Large impact type. **(A)** The ratio density of cumulative mentions for the four time intervals. The time interval setting is same as Transient type. **(B)** The Q-Q plots of the distribution of cumulative mentions relative to the normal distribution.

**Figure 8:**
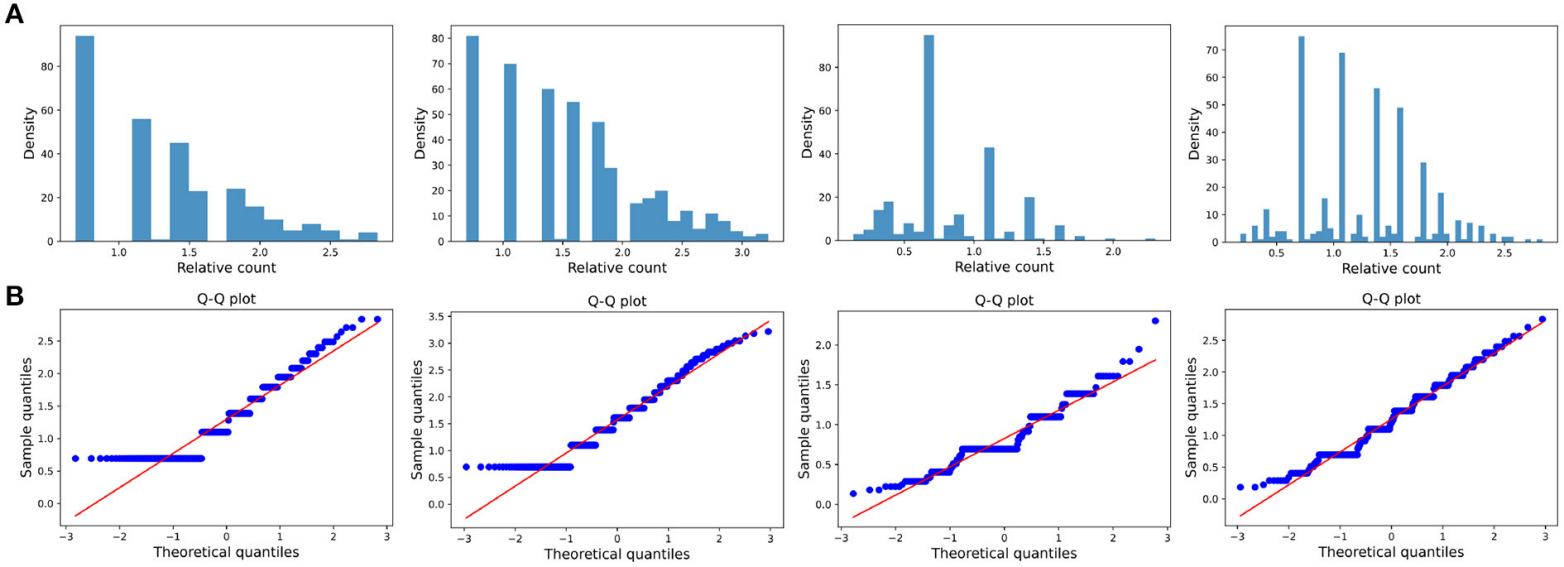
Small impact type. **(A)** The ratio density of cumulative mentions for the four time intervals. The time interval setting is same as Transient type. **(B)** The Q-Q plots of the distribution of cumulative mentions relative to the normal distribution.

The distribution of the ratios *C*_*k*_(*t*_*i*_, *t*_*j*_) seems to fit the log-normal distributions well (Figures 6A, 7A, 8A). We rescale the horizontal axis logarithmically and find that the histogram roughly shows the form of a Gaussian function. To verify that the above assumption is mathematically correct, we perform a Q-Q plot and KS test for verification. The Q-Q plot of the normal distribution is a scatter plot with the quantile of the standard normal distribution as the *x* and the sample value as the *y*, the scatter plot obtained from the normal distribution should be a straight line (Figures 6B, 7B, 8B). In addition, from the Q-Q plot, we found that the closer to the current time period, the more obvious its normal distribution is.

We also test whether we can accept the null hypothesis that the sample distribution is normally distributed by comparing the critical value of the 99% confidence with the KS statistic for each fixed time horizon. As the results shown in Tables 3–5, in almost all of the cases, we accept the null hypothesis that the ratio of cumulative mentions of keywords exhibit a normal distribution. However, it should be noticed that, for the Small impact type, the distributions in (2010, 2015) and (2010, 2020) fail to pass the KS test, which means there exists some relatively large divergence in the number of mention times.

**Table 3:** KS test of Transient type: the critical value of the KS statistic at 99% confidence and the KS statistic for each fixed time horizon.

	**(2000, 2015)**	**(2000, 2020)**	**(2010, 2015)**	**(2010, 2020)**
Number of samples	5	12	6	12
99% confidence threshold	0.669	0.450	0.618	0.450
KS statistic	0.322	0.326	0.407	0.390

**Table 4:** KS test of Large impact type: the critical value of the KS statistic at 99% confidence and the KS statistic for each fixed time horizon.

	**(2000, 2015)**	**(2000, 2020)**	**(2010, 2015)**	**(2010, 2020)**
Number of samples	21	22	17	27
99% confidence threshold	0.349	0.342	0.381	0.306
KS statistic	0.213	0.282	0.250	0.217

**Table 5:** KS test of Small impact type: the critical value of the KS statistic at 99% confidence and the KS statistic for each fixed time horizon.

	**(2000, 2015)**	**(2000, 2020)**	**(2010, 2015)**	**(2010, 2020)**
Number of samples	16	24	31	52
99% confidence threshold	0.392	0.328	0.283	0.226
KS statistic	0.253	0.236	0.331	0.336

There are numbers of studies explaining the emergence of the log-normal distribution. Mitzenmacher (2004) pointed out that the emergence of lognormal is the result of a multiplicative growth process with noise. Embrechts indicated that the log-normal distribution of the keyword occurrence distribution can be explained by a simple stochastic dynamical model (Wu and Huberman, 2007). In this study, when a new research direction or hotspot emerges, it first attracts the attention of a small number of researchers. If they think the emergence of these discussion points is meaningful enough, they may spread these hotspots further. With the development of time, if more researchers start to pay attention to this research point, then this hotspot will continue to increase its popularity in this field. In other words, a positive reinforcement effect indicates that the more popular a hotspot becomes, the faster it spreads (Wu and Huberman, 2007). But as time goes by, the popularity of the discussion hotspot will become less and less. Because more and more researchers are aware of these discussion points, they will no longer be a research hotspot.

Based on the above discussion, here we introduce a process known as the random multiplicative noise process with growth to explain the keywords' evolution. In this model, the increment in the number of cumulative mentions in the next defined interval can be expressed as the cumulative number of mentions in the previous interval multiplied by a random number. Define *N*_*k*_(*t*) as the cumulative mentions of keyword *k* over time interval *t*. It can be known that *N*_*k*_(*t*) will only increase rather than decrease with time. This process can be expressed as,


(9) $$\begin{array}{c} N_{k}\left(t\right)=\left[1+\xi \left(t\right)\right]N_{k}\left(t-1\right), \end{array}$$

where ξ(*t*) is positive and independent identically distribution as a function of *t* with mean μ and variance σ^2^. ξ(*t*) is positive, ensuring that *N*_*k*_(*t*) can only grow over time.

But the novelty of keywords tends to fade over time, so researchers' attention to them also fades away. We parameterize by defining a factor γ(*t*) consisting of a series of decreasing positive numbers. The dynamical model of the log-normal distribution over the keyword *k* can be expressed as,


(10) $$\begin{array}{c} N_{k}\left(t\right)=\left[1+\gamma \left(t\right)\xi \left(t\right)\right]N_{k}\left(t-1\right), \end{array}$$

γ(*t*)ξ(*t*) is called as discounted random multiplicative factor (Wu and Huberman, 2007).

The above equation will results in a log-normal distribution. Here, the *N*_*k*_(*t*) is the initial number of mentions in the time step, the right side of the Equation (12) is the sum of a large number of random variables. The central limit theorem (Embrechts and Maejima, 1984) indicates that if random variables are independent identically distributed, then the sum will asymptotically approximated to a normal distribution.


(11) $$\begin{array}{l} \begin{array}{l} N_{k}\left(t\right)=\left[1+\gamma \left(t\right)\xi \left(t\right)\right]N_{k}\left(t-1\right)\\ \;=\overset{t}{\underset{s=1}{\prod }}\left[1+\gamma \left(s\right)\xi \left(s\right)\right]N_{k}\left(0\right), \end{array} \end{array}$$

(12) $$\begin{array}{c} lnN_{k}\left(t\right)-lnN_{k}\left(0\right)=\overset{t}{\underset{s=1}{\sum }}ln\left[1+\gamma \left(s\right)\xi \left(s\right)\right]. \end{array}$$

#### 3.2.2. The novelty of keywords

In order to measure the functional form of γ(*t*), if we set the expected value of noise term ξ as 1 (Asur et al., 2011) in Equation (12), the novelty variation of keywords can be easily obtained by some simple algebra, that is, γ(*t*) can be calculated by averaging the ratios between cumulative mentions of keywords in the current year *t* and in the last year *t* − 1.


(13) $$\begin{array}{c} \gamma \left(t\right)=<\frac{N_{k}\left(t\right)}{N_{k}\left(t-1\right)}{>}_{k}-1. \end{array}$$

**Figure 9:**
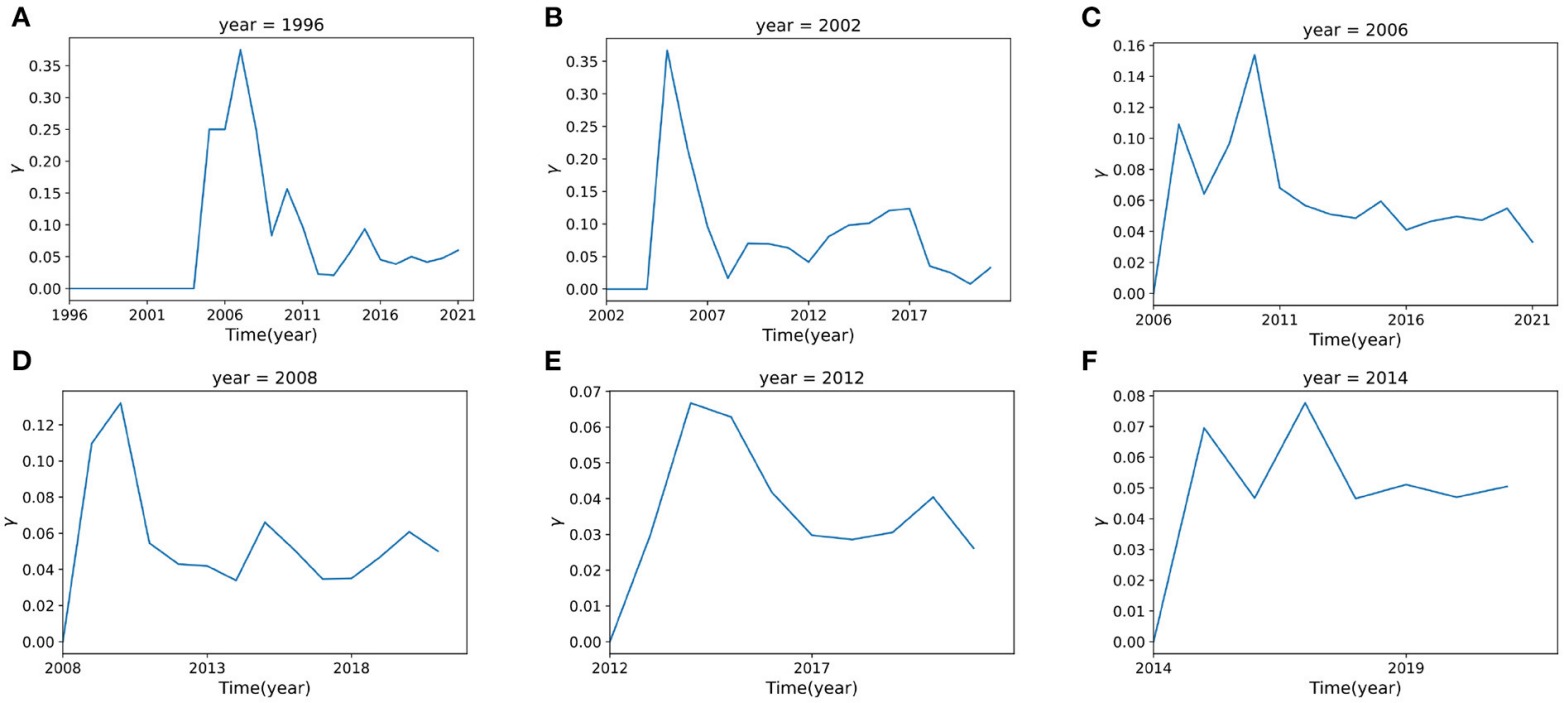
The novelty factor γ(*t*) with time. **(A–F)** represent the curves of γ(*t*) for time *t* = 1996, 2002, 2006, 2008, 2012, 2014 respectively. The curve of γ(*t*) is fitted from the data which are calculated from Eq. 12.

To illustrate γ(*t*), we make some examples for different years. For instance, there are 4 keywords that first appeared in 1996, which are language, single trial, prefrontal and neuroimaging. Figure 9A shows the change of novelty of these keywords since 1997. In 1997, researchers paid no attention to these four keywords, γ(1997) = 0. This situation didn't change until *t* = 2005, when there's a sharp increase of γ(*t*). However, this trend did not last long, after the year 2007, γ(*t*) began to decrease gradually.

Figure 9 shows the novelty change γ(*t*) for all keywords that first appeared in 1996, 2002, 2006, 2008, 2012, and 2014. It can be found that the novelty of keywords presents similar patterns. They all attract attention at the beginning, with the corresponding novelty value increasing to some peak, however, after the peak point, they always tend to decrease rapidly, indicating a loss of interest.

### 3.3. Elite keywords

**Figure 10:**
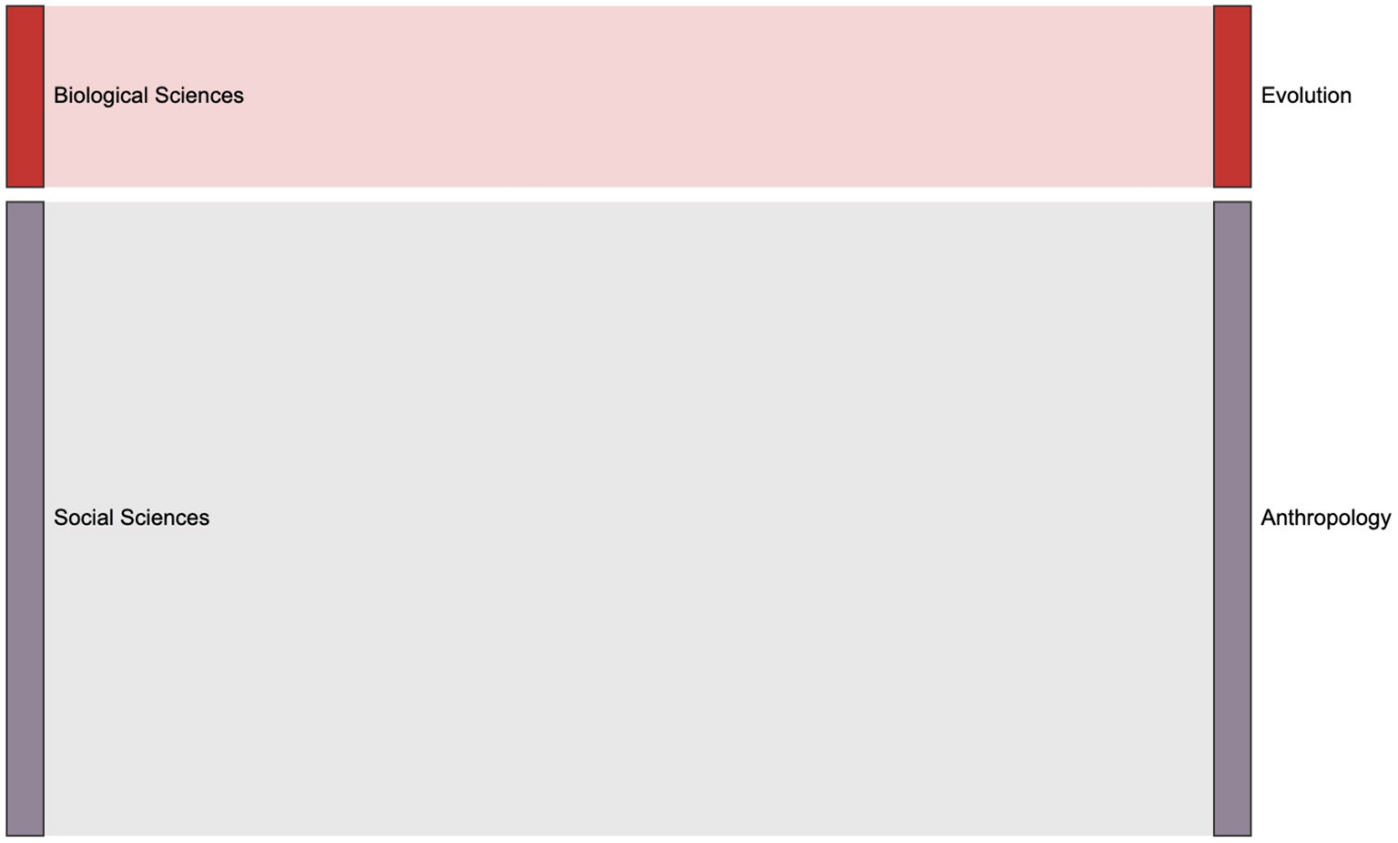
The distribution of keyword mention times in various fields in the 1990s, the left column is the first-level scientific field including Biological Sciences, Social Science and Physical Science, and the right class is the second level Segmentation field.

**Figure 11:**
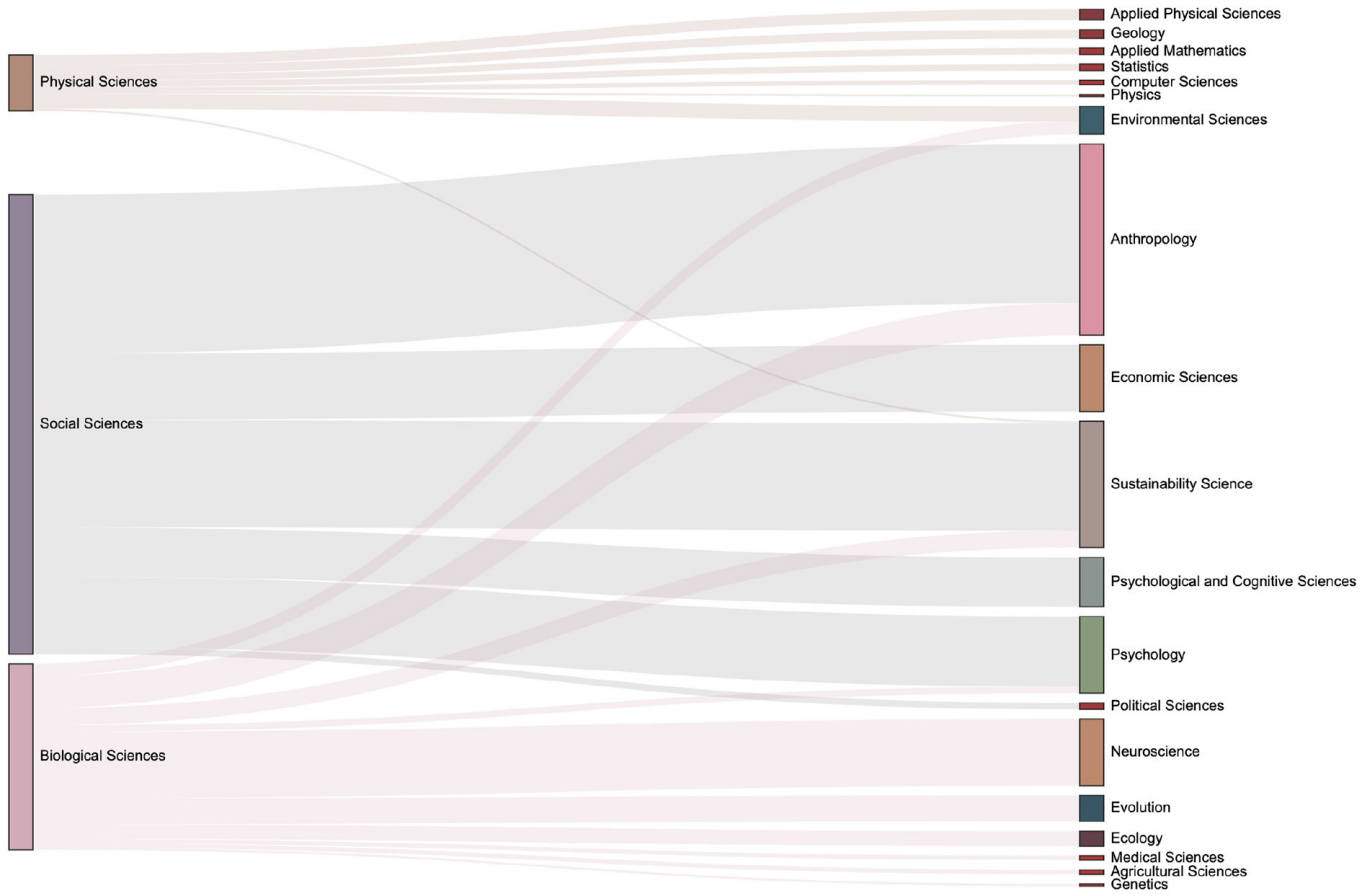
The distribution of keyword mention times in various fields in the 2000s. It can be seen that more than 20 subfields have emerged during 2000s.

**Figure 12:**
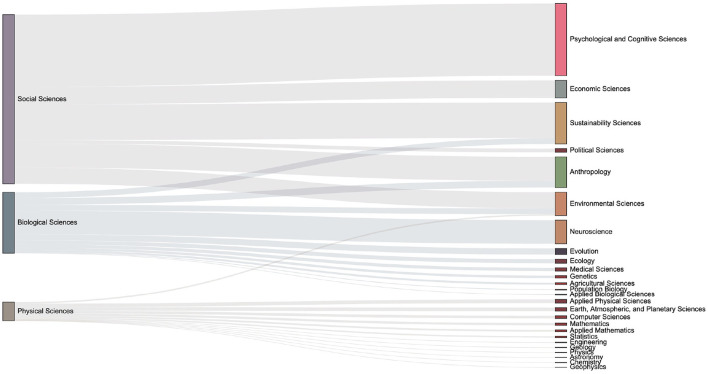
The distribution of keyword mention times in various fields in the 2010s. One can observe the number of articles in each sub-category from the size of the box on the right column. Compared with the previous period, it can be seen that there are increasing discussions related to the Environmental Sciences.

**Figure 13:**
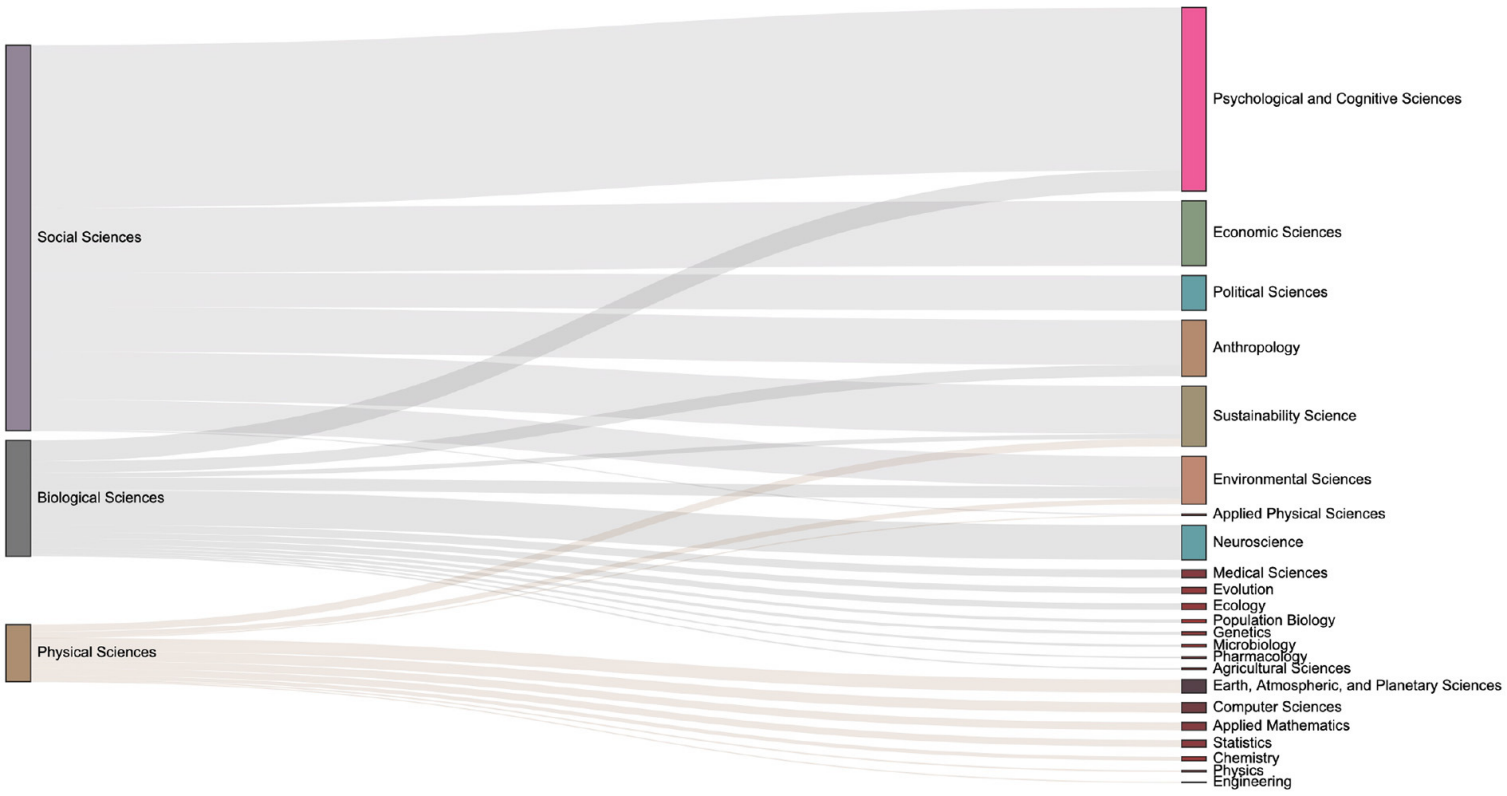
The distribution of keyword mention times in various fields in the 2020s. Compared with the previous period, discussions on economic-related issues have increased during this period.

The data set used in this paper contains the domain concepts which are assigned by journal. Articles have the first-level fields such as Physical Sciences, Social Sciences, and Biological Sciences, and more subdivided second-level fields such as Economic Sciences, Psychology, et al.. We introduce the Sankey diagram to show the proportion of the output of each field in each period. The size ratio of the bars on the right side of the Figures 10–13 corresponds to the ratio of scientific output.

**Figure 14:**
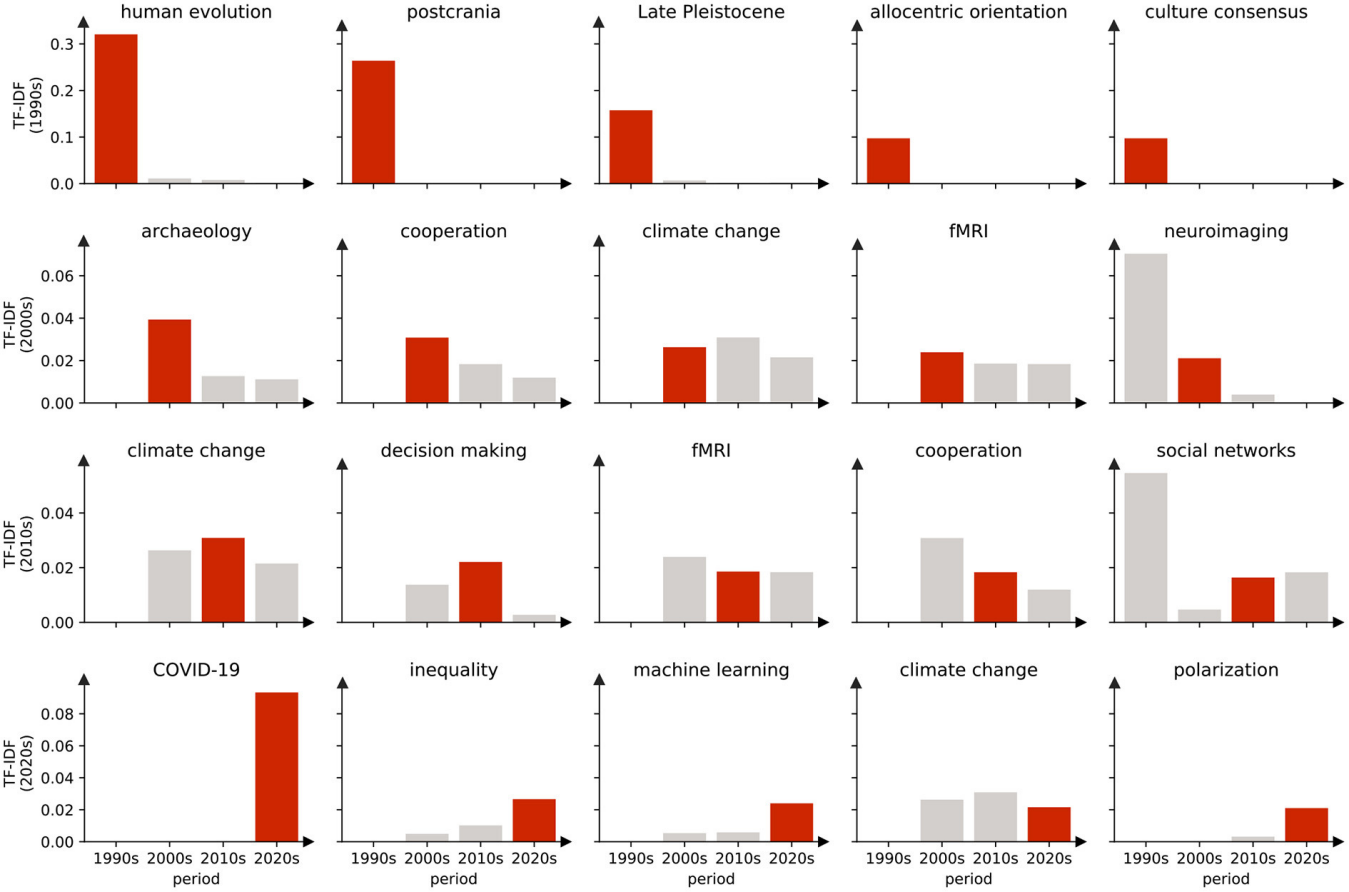
Top five elite keywords for each era, most of which have the highest TF-IDF values in their corresponding eras. For example, the TF-IDF values of the five keywords in the 1990s obviously have the maximum value in the 1990s, and are almost all 0 in the 2000s and after, corresponding to these values only important for the 1990s. But there were also keywords that were represented as elite keywords in more than one era. For example, climate change, which was classified as an elite keyword in the 2000s, 2010s, and 2020s, is an area of concern.

We defined elite keywords which have the greatest impact on the research direction or content of each period. Expressed in a statistical language, we assign a ranking value to each keyword according to the TF-IDF value as discussed previously. A larger TF-IDF value of a keyword indicates a larger importance of the keyword in this period. By ranking the TF-IDF of each keyword in an era from large to small, the top words are considered as the elite keywords in a certain era. As shown in Figure 14, one can see the corresponding TF-IDF value distribution of the top five elite keywords in each era. It can be found that most of the elite keywords collected in the corresponding era have the largest TF-IDF values.

The top 20 elite keywords in the field of social sciences are listed in Table 6, which also includes the percentage of publications which the keyword belongs to. During the 1990s, researchers mainly focus on the perspectives related to human evolution, postcrania and Late Pleistocene. During the 2000s, topics change to archaeology, cooperation, climate change, fMRI, and neuroimaging. During the 2010s, the researchers focus on climate change, decision making, fMRI, cooperation, and social networks. In the 2020s, the focus turned to COVID-19, inequality, machine learning, climate change, and polarization. In fact, the dominant keywords have always been changing over time.

**Table 6:** Top 20 elite keywords in each era, and the proportion of publications that contain elite keywords.

**1990s**			**2000s**			**2010s**			**2020s**		
**Keyword**	**TF-IDF**	**%**	**Keyword**	**TF-IDF**	**%**	**Keyword**	**TF-IDF**	**%**	**Keyword**	**TF-IDF**	**%**
Human evolution	0.32	25.00	Archaeology	0.04	5.23	Climate change	0.03	4.13	COVID-19	0.09	12.61
Postcrania	0.26	15.00	Cooperation	0.03	3.86	Decision making	0.02	2.42	Inequality	0.03	5.17
Late Pleistocene	0.16	10.00	climate change	0.03	3.18	fMRI	0.02	2.15	Machine learning	0.02	2.43
Allocentric orientation	0.10	5.00	fMRI	0.02	3.18	Cooperation	0.02	2.09	Climate change	0.02	3.19
Culture consensus	0.10	5.00	Neuroimaging	0.02	1.82	Social networks	0.02	1.76	Polarization	0.02	2.74
DNA diversity	0.10	5.00	Emotion	0.02	3.18	Culture	0.01	1.32	fMRI	0.02	2.43
Japanese–English comparisons	0.10	5.00	Agriculture	0.02	2.50	Archaeology	0.01	1.38	Social networks	0.02	1.98
Magnetic source imaging	0.10	5.00	Cultural transmission	0.02	1.36	Social cognition	0.01	1.16	Mortality	0.02	2.74
Cognitive models	0.10	5.00	Game theory	0.02	2.27	Aging	0.01	1.10	Decision making	0.02	1.67
Maximum likelihood method	0.10	5.00	Neolithic	0.02	2.05	Attention	0.01	1.10	Gender	0.02	2.74
Granule cells	0.10	5.00	Development	0.02	5.23	Sustainability	0.01	1.10	Public health	0.02	1.67
Sensitivity analysis	0.10	5.00	Vision	0.02	1.59	Field experiment	0.01	0.99	Social media	0.02	1.67
Path integration	0.10	5.00	Memory	0.02	3.86	Agriculture	0.01	1.05	Language	0.02	3.95
Brownian motion	0.10	5.00	Cognitive development	0.02	1.36	Emotion	0.01	0.99	Misinformation	0.02	1.22
Neutrality	0.10	5.00	Institutions	0.02	1.59	Neuroeconomics	0.01	0.94	Culture	0.01	3.34
Ancestral polymorphism	0.10	5.00	Domestication	0.02	1.59	Inequality	0.01	1.05	Demography	0.01	1.52
Silent substitution rate	0.10	5.00	Phylogeography	0.01	0.91	Evolution	0.01	0.94	Vaccination	0.01	1.52
Black–Scholes–Merton theory	0.10	5.00	Decision making	0.01	1.82	Cognition	0.01	0.94	Education	0.01	2.74
Population forecasting	0.10	5.00	Evolution	0.01	7.27	Behavioral economics	0.01	0.88	Hippocampus	0.01	1.22
Insularity	0.10	5.00	Syntax	0.01	0.91	Game theory	0.01	0.88	Uncertainty	0.01	1.22

The sorting of elite keywords is first sorted according to the TF-IDF value from large to small, and then the keywords with the same TF-IDF value are sorted according to the TF value. If the TF-IDF value of the keyword and the TF value are the same, then sort according to the IDF.

Since PNAS may assign each article with several sub-field tags, each of the above-extracted elite keywords may also be assigned with the same sub-fields tags. To avoid confusion we choose the sub-field in which the keyword has the most tags as the main subject area to which the keyword belongs. For example, the keyword public health appears 24 times in the sub-field of Social Sciences and 7 times in the sub-field of Biological Sciences, and we choose to tag it with the sub-field of Social Sciences. In this sense, we make a statistic of all the main subject areas for the 80 elite keywords listed in Table 6. We find that all these elite keywords can be classified into 8 subject areas, which are Social Sciences, Psychological and Cognitive Sciences, Biological Sciences, Sustainability Sciences, Anthropology, Physical Sciences, Neuroscience, and Environmental Sciences. Besides, we classify the keywords which do not have assigned sub-field tags into the subject area “Methods” as we named. In this sense, we have nine broad categories as shown in the left column of Table 7.

**Table 7:** List of nine subject areas and the corresponding elite keywords.

**Areas**	**Keywords**
Social Sciences	Uncertainty, public health, vaccination, social media, misinformation, behavioral economics, cultural transmission, evolution, aging, syntax, COVID-19, mortality, language, inequality, education, development, institutions, cooperation, gender, culture
Psychological and cognitive sciences	Attention, hippocampus, allocentric orientation, social cognition, decision making
Biological sciences	Granule cells, DNA diversity
Sustainability science	Sustainability, agriculture
Anthropology	Postcrania, DNA diversity, domestication, culture consensus, human evolution, neutrality, demography, evolution, Late Pleistocene, silent substitution rate, ancestral polymorphism, archaeology, Japanese–English comparisons, Neolithic, insularity, phylogeography, population forecasting
Physical sciences	Brownian motion, polarization
Neuroscience	Cognition, memory, neuroimaging, emotion, cognitive development, fMRI, neuroeconomics, vision, magnetic source imaging
Environmental sciences	Climate change
Methods	Sensitivity analysis, field experiment, game theory, social networks, path integration, cognitive models, Black–Scholes–Merton theory, maximum likelihood method, machine learning

**Figure 15:**
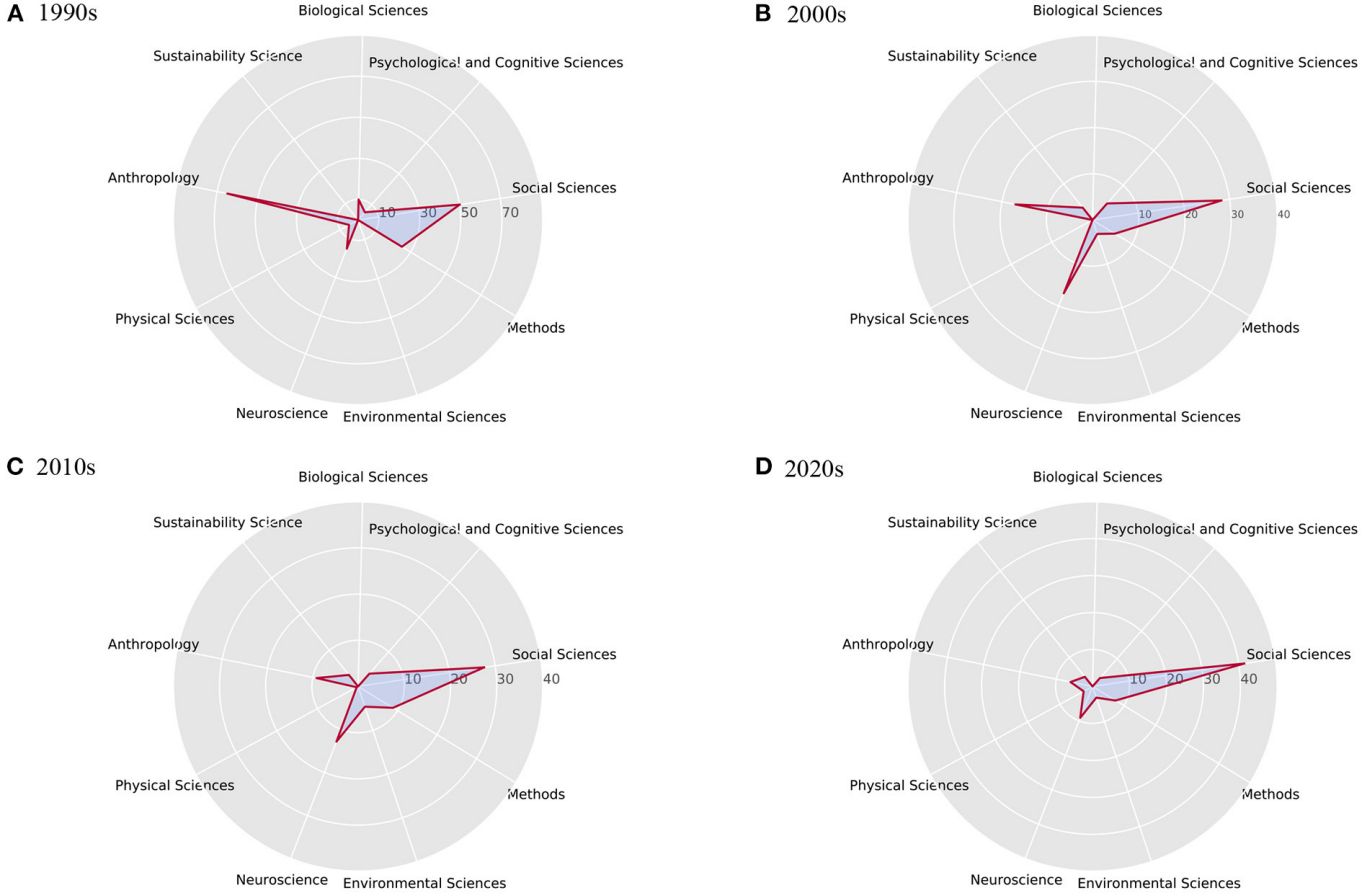
Corresponding percentage of publications related to nine broad topic for four eras. It can be seen that in the 1990s **(A)**, many researchers focused on anthropology. In the 2000s **(B)**, neuroscience became a hot topic. While in the 2010s **(C)** and 2020s **(D)**, research in the field of social sciences mostly focus on social problems and scientific solving methods.

For each era, we also make a statistic of the article numbers corresponding to the above nine topics, which is shown in Figure 15 as radar diagrams. Research hotspots reflected by article numbers can be observed to change from anthropology research to neuroscience and social problems research from the evolution diagram.

In the 1990s, about 65% of the articles contained keywords related to anthropology, and about 25% of the articles contained evolution-related content. The theory of social evolution is one of the main ideas of modern social sciences. Much of the history of modern social sciences can be described by the twists and turns that mark the history of social evolutionary theory, which was very popular in the 1990s (Harvey and Reed, 1994). The elite keywords extracted during this period did not reappear in the next three generations of elite keywords, which can clearly reflect the research hotspots in this period. During this period, researchers mostly carried out research in related fields from two major categories of biological sciences and social sciences, and a major field of anthropology.

Compared with 1990s, the obvious difference in the 2000s is the relatively large proportion of neuroscience-related publications, reaching 17.05%. Neuroscience has become a hot topic in assisting social sciences research from the perspective of biological behavior and psychology. Social phenomena can be described and explained through different levels of analysis, which correspond to theories and methodologies of different disciplines. The emergence of collective social phenomena is based on individual psychological processes, which in turn depend on brain processes (Antonietti and Iannello, 2011). Another notable difference is the focus on climate change, which has clear human, social and cultural drivers (Zhang et al., 2007). Many scientists have warned of the social risks that climate change may bring and conducted research on the impact of climate change on society (Polyak and Asmerom, 2001; Mann and Jones, 2003).

In the 2010s, the fields of neuroscience and social sciences are still the two most popular fields. The keyword climate change ranks first among elite keywords. It was only during this period that scientists began to believe that changes in the world's climate were the new specter plaguing the planet (Urry, 2015). Climate change is not a purely scientific issue, human and social behavior is at the heart of the apparent warming of the planet. Scientific and technological innovations are necessary, but making them impact requires understanding how people adapt and change their behavior (Shah, 2020). From the extracted elite keywords, it can also be observed that many research directions related to applied mathematics and computational social sciences have emerged during this period.

In the 2020s, the event that caught the attention of researchers most during this period was the emergence of COVID-19, followed by concerns about inequality. We cannot improve global health if we only view medicine narrowly (Shah, 2020). Epidemics are not only biological phenomena, but also social phenomena. Pandemic create and exacerbate financial and employment inequalities that affect people's mental health and have social consequences (Kousoulis et al., 2020). Research on inequality requires not only mathematical methods to solve, but also the involvement of professional knowledge in the social field.

By extracting the elite keywords of each period and tracking the changes of the elite keywords of the era, we get the focus distribution of the field era. With the development of science and technology, the methods and research perspectives in the field of social sciences have undergone tremendous changes, and many interdisciplinary fields have emerged. It means that the problems existing in social phenomena cannot be solved only from one perspective, but need to be comprehensively dealt with knowledge from multiple perspectives and fields.

## 4. Discussion

The evolution pattern of keywords is an efficient indicator in revealing the shifting and sustainability configuration of scientific concepts, ideas, and research hotspots. Here we take an extensive investigation of the evolution of keywords among all publications in PNAS Social Sciences topic from 1990 to 2021.

A novel schema of four patterns (TELS) is proposed to illustrate the evolution of keywords. The TELS schema can be used to capture the whole life circle feature of any proposed keyword, from a pool of candidates. Our research showed that most keywords in the field are only short-lived in history, and just a little part of keywords has long-term effects on the field. Moreover, statistical tests show that a log-normal distribution fits most keywords. We represent the number of keyword mentions with a simple stochastic dynamical model and find that keyword novelty has a decay characteristic. We also introduce the concept of elite keywords to reveal the temporal feature of social sciences focus. An explicit transition from anthropology and evolution research to neuroscience and social problems research can be observed from the evolution diagram.

Different journals in the same field may have similar research perspectives or research methods for the same issue, or there may be completely different perspectives and methods. In this study, we only perform keyword analysis for articles published in the field of PNAS Social Sciences between 1990 and 2021. In the future, it may be useful to study the keyword evolution of all articles published in other journals in the field of social sciences, so as to discover the general research interest and help researchers choose appropriate journals from the perspective of content. We argue that the proposed method is general and might be applicable to other fields of science.

## Data availability statement

The raw data supporting the conclusions of this article will be made available by the authors, without undue reservation.

## Author contributions

BL: data curation, investigation, methodology, and writing—original draft. MS and YK: methodology and writing. XJ: supervision, conceptualization, and writing—review and editing. All authors contributed to the article and approved the submitted version.

## Funding

This work has been supported by Guangxi Province Science and Technology Program (2021AA11006), Beijing Natural Science Foundation (Z180005), and the Fundamental Research Funds for the Central Universities (YWF-22-L-639).

## Conflict of interest

The authors declare that the research was conducted in the absence of any commercial or financial relationships that could be construed as a potential conflict of interest.

## Publisher's note

All claims expressed in this article are solely those of the authors and do not necessarily represent those of their affiliated organizations, or those of the publisher, the editors and the reviewers. Any product that may be evaluated in this article, or claim that may be made by its manufacturer, is not guaranteed or endorsed by the publisher.
